# Cardiovascular disease risk communication in NHS Health Checks: a qualitative video-stimulated recall interview study with practitioners

**DOI:** 10.3399/BJGPO.2021.0049

**Published:** 2021-08-04

**Authors:** Christopher J Gidlow, Naomi J Ellis, Victoria Riley, Lisa Cowap, Diane Crone, Elizabeth Cottrell, Sarah Grogan, Ruth Chambers, Sian Calvert, David Clark-Carter

**Affiliations:** 1 Centre for Health and Development, School of Life Sciences and Education, Staffordshire University, Stoke-on-Trent, UK; 2 Cardiff School of Sport and Health Sciences, Cardiff Metropolitan University, Cardiff, UK; 3 School of Primary, Community and Social Care, Keele University, Keele, UK; 4 Department of Psychology, Manchester Metropolitan University, Manchester, UK; 5 Stoke-on-Trent Clinical Commissioning Group, Stoke-on-Trent, UK

**Keywords:** cardiovascular diseases, risk, preventive medicine, primary health care, qualitative research

## Abstract

**Background:**

NHS Health Check (NHSHC) is a national programme to identify and manage cardiovascular disease (CVD) risk. Practitioners delivering the programme should be competent in discussing CVD risk, but there is evidence of limited understanding of the recommended 10-year percentage CVD risk scores. Lifetime CVD risk calculators might improve understanding and communication of risk.

**Aim:**

To explore practitioner understanding, perceptions, and experiences of CVD risk communication in NHSHCs when using two different CVD risk calculators.

**Design & setting:**

Qualitative video-stimulated recall (VSR) study with NHSHC practitioners in the West Midlands.

**Method:**

VSR interviews were conducted with practitioners who delivered NHSHCs using either the QRISK2 10-year risk calculator (*n* = 7) or JBS3 lifetime CVD risk calculator (*n* = 8). Data were analysed using reflexive thematic analysis.

**Results:**

In total, nine healthcare assistants (HCAs) and six general practice nurses (GPNs) were interviewed. There was limited understanding and confidence of 10-year risk, which was used to guide clinical decisions through determining low-, medium-, or high-risk thresholds, rather than as a risk communication tool. Potential benefits of some JBS3 functions were evident, particularly heart age, risk manipulation, and visual presentation of risk.

**Conclusion:**

There is a gap between the expectation and reality of practitioners’ understanding, competencies, and training in CVD risk communication for NHSHCs. Practitioners would welcome heart age and risk manipulation functions of JBS3 to promote patient understanding of CVD risk, but there is a more fundamental need for practitioner training in CVD risk communication.

## How this fits in

CVD risk assessment and communication is central to NHSHCs. Knowledge around practitioners’ associated understanding, confidence, and perceptions, and the potential benefit of using newer CVD risk calculators, could inform changes to improve NHSHC delivery. This study highlights a mismatch between expected practitioner competencies and training in CVD risk communication in NHSHCs and the reality. The study reports limited understanding and utility of 10-year risk as a risk communication tool, and potential benefits of heart age and risk manipulation functions of JBS3. However, there is a more fundamental need for practitioner training.

## Introduction

The NHSHC programme was established to prevent CVD in adults in England aged 40–74 years.^1^ The most common delivery setting is primary care where practitioners, usually GPNs or HCAs, measure the patient’s CVD risk, communicate those results to the patient, discuss CVD risk management, and are expected to have associated training and competencies.^2,3^

In keeping with National Institute for Health and Care Excellence (NICE) guidance, standard practice is to assess CVD risk using QRISK2 (or QRISK3), which estimates a patient’s percentage risk of having a heart attack or stroke in the next 10 years.^4^ As Bonner *et al* ^5^ noted, such absolute CVD risk estimates were not developed as tools to promote patient understanding, but to guide clinical decision making (for example, to discuss statins where 10-year risk ≥10%).^4^

**Figure 1.**
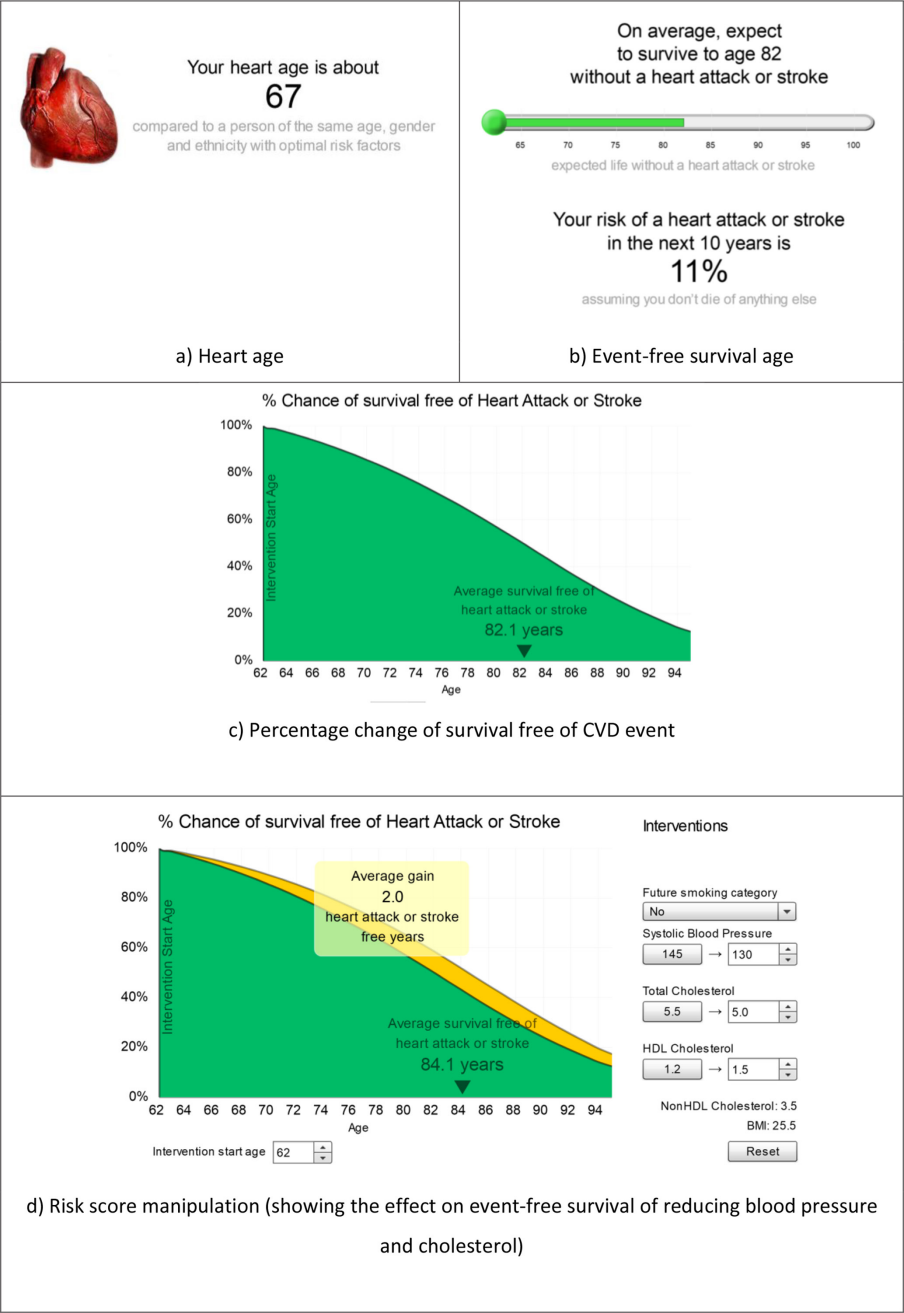
Example of Joint British Societies for the prevention of cardiovascular disease (JBS3) outputs.^11^ Images borrowed with permission from JBS3. BMI = body mass index. CVD = cardiovascular disease. HDL = high-density lipoprotein.

Evidence that patients and practitioners have limited understanding of such risk scores^6–9^ turned attention towards alternative CVD risk metrics. In 2014, the Joint British Societies for the prevention of CVD (JBS) launched the JBS3 risk calculator^10,11^ with a primary focus on lifetime CVD risk. JBS3 includes several CVD risk metrics and functions to address limitations of short-term, absolute risk estimates (for example, underestimation of risk in younger adults and interpreting percentages), and facilitate patient understanding and decision making about CVD risk management (Figure 1). Features include:

- *Heart age:* the estimated age of someone of the same sex, ethnic group, and risk of an annual event, but with all other CVD risk factors at ‘optimal’ levels (Figure 1a).^10^ Someone with a comparatively ‘old’ heart age should be motivated to undertake behaviour that can reduce it towards their chronological age. Evidence suggests that heart age is more easily communicated to, understood, and recalled by patients.^5,12^ A rapid review that included four randomised controlled trials (RCTs) of change in lifestyle behaviour or risk factors following risk communication using heart (or cardiovascular) age versus absolute risk or ‘usual care’, reported outcomes that generally favoured heart age (statistically or clinically significant), but noted concerns about study quality.^13^ There is some evidence of benefit for clinical risk factor management when heart age is combined with other components,^14,15^ but overall, randomised studies directly comparing heart age with percentage risk do not show that heart age is a motivating risk format.^16^ Moreover, potential limitations of heart age include perceived credibility, negative emotional response, and inflated risk perception.^5,17^

- *Event-free survival age:* the age by which an individual might expect to have their first CVD event, based on current risk and demographic profile. JBS3 presents this as a visual analogue scale, stating that the user can *‘on average, expect to survive to age XX without a heart attack or stroke* *‘* (Figure 1b).
- *Percentage chance of survival free of CVD event:* a survival curve, which illustrates the decreasing chance of being free of a heart attack or stroke with increasing age (based on current risk and demographic profile; Figure 1c).
- *Risk score manipulation:* explicitly possible in JBS3 through modifying risk factors (for example, smoking status, blood pressure, and cholesterol) to demonstrate how intervention can reduce CVD risk. Such interactive graphics can be beneficial through engaging individuals with the information, promoting understanding, and retention^18,19^ (Figure 1d).
- *Visual displays*: a variety of icon arrays or Cates plots, an image of a heart for heart age, visual analogue scales, and survival curves aim to accommodate a range of patient needs and preferences,^18^ and may promote risk-reducing behaviour.^20^

Health professionals’ experiences of the NHSHC programme have been explored, but not focusing on CVD risk communication. A 2017 review included 10 studies reporting the views of health professionals in primary care^21^ and identified scepticism regarding the effectiveness of NHSHCs in moving patients at high-risk towards risk-reducing behaviours. However, few captured views of those delivering the NHSHCs and none specifically considered CVD risk communication. A study of 38 videorecorded NHSHCs found that QRISK2 was communicated in over 97% of cases, but did not report the extent of risk discussion or practitioner understanding.^22^

This article presents data from VSR interviews with NHSHC practitioners from the RIsk COmmunication in NHSHC (RICO) study.^23^ RICO involved analysis of videorecorded NHSHCs to understand how CVD risk was communicated when using QRISK2 or JBS3. Published findings from the RICO study suggest that: practitioners spend little time discussing CVD risk overall, although slightly more when using JBS3 (compared with QRISK2); CVD-risk discussions tend to be practitioner-dominated and more information-giving than dialogue;^24,25^ and practitioners often miss opportunities to engage patients in risk discussion, perhaps indicating a lack of confidence.^25^ In the RICO study, VSR interviews with practitioners were used to explore underlying reasons. VSR interviews are well-suited to study complex clinician–patient interactions,^26,27^ described as going beyond fact-finding and description to generate more meaningful explanations of events in consultations.^28^ They have been used in primary care to study the discussion of various health topics (for example, osteoarthritis and preventive services)^27^ and aspects of consultations (for example, patient versus clinical perspectives, patient response, and communication).^26,28^

This article reports findings from VSR interviews with practitioners from the RICO study. Excerpts from videorecorded NHSHCs were used in interviews to prompt recall and reflection, with the aim of exploring practitioner understanding, perceptions, and experiences of CVD risk communication in NHSHCs using QRISK2 or JBS3 CVD risk calculators.

## Method

### Setting and participants

Data were collected as part of the RICO study. Study processes are reported in detail elsewhere.^23^ RICO involved 12 general practices in the West Midlands of England recruited through the Clinical Research Network. Practice pairs matched by deprivation^29^ were randomised to continue using QRISK2 to communicate CVD risk in NHSHCs (usual practice), or to use the JBS3 CVD risk calculator following brief introductory training (intervention). Each practice was asked to videorecord NHSHCs until 20 useable consultations were recorded. Two practices allocated to ‘usual practice’ used additional software (Informatica), which had some JBS3 functionalities (for example, heart age and risk manipulation). These data were included as this reflected their usual practice and relevant data are highlighted using the label QRISK2+.

Participants were, therefore, a purposive sample of all 15 primary care practitioners (nine HCAs and six GPNs) who delivered NHSHCs within the 12 RICO general practices (Table 1). They were invited to take part in interviews during practice initiation visits.

**Table 1.** Practitioner characteristics

Practice	Risk calculator	PID	Role	Sex	Ethnic group	Time delivering NHSHC	NHSHC training	Recorded NHSHC, *n*	Interview duration, min
1	JBS3	1.1	GPN	F	WBRI	9 years	No formal training	7	72.2
		1.2	HCA	F	WBRI	6 years	Generic, PoC training	5	65.3
2	QRISK2	2.1	HCA	F	WBRI	2.5 years	Generic training	22	47.4
3	QRISK2	3.1	HCA	F	WBRI	2.5 years	No formal training(at time of study)	14	28.8
4	JBS3	4.1	HCA	F	Ethnic minority	2 years	No formal training	29	47.1
5	JBS3	5.1	GPN	F	WBRI	8 years	Generic training x 2	7	40.3
6	QRISK2	6.1	GPN	F	WBRI	2 years	No formal training	6	36.3
		6.2	GPN	F	WBRI	6 years	Generic, lifestyle advice and referrals	11	50.2
7	JBS3	7.1	HCA	F	WBRI	5 years	Generic, PoC training(could not recall details)	20	66.2
8	JBS3	8.1	HCA	F	WBRI	5 years	Generic training	11	84.0
		8.2	GPN	F	WBRI	9 months	No formal training	13	58.6
9	QRISK2	9.1	HCA	F	WBRI	6 years	Generic training(could not recall details)	5	45.5
10	QRISK2	10.1	GPN	F	WBRI	3 years	No formal training	3	45.6
11	JBS3	11.1	HCA	F	Ethnic minority	8 years	Generic x 2(8 and 1 years earlier)	8	53.2
12	QRISK2	12.1	HCA	F	WBRI	4 years	Generic(4 years earlier)	12	48.9

GPN = general practice nurse. HCA = healthcare assistant. JBS3 = Joint British Societies for the prevention of cardiovascular disease. NHSHC = NHS Health Check. PID = personal identifier. PoC training = trained to use the point-of-care testing machine. WBRI = White British.

### Procedures

Semi-structured one-to-one VSR interviews were conducted at the general practice, within 2 weeks of the practitioners’ final recorded NHSHC. After each clinic, two researchers (VR and LC) viewed recorded NHSHCs to identify sections of the consultation to use in VSR interviews. Sections were selected if they featured discussion of the CVD risk score, manipulation of the risk score (in the JBS3 group), or provision of advice, recommendations, and interventions by the practitioner. Interviews followed a pre-piloted process and topic guide, tailored to QRISK2 or JBS3 groups. Practitioners in the QRISK2 group were shown JBS3 outputs with accompanying explanation and asked to comment, and JBS3 participants had experience of QRISK2 so were able to comment on both risk calculators; thus, practitioners from both groups commented on both CVD risk calculators.

Two White British female researchers with extensive interview experience conducted interviews: a qualified health psychologist and lecturer in health psychology (LC); and a research associate with a background in health psychology and NHSHC research (VR). Before the study, the researchers did not have any relationship with participants. No others (that is, non-participants) were present during interviews, which were audiorecorded and transcribed verbatim for analysis.

### Analysis

Patient VSR interview transcripts were analysed using inductive reflexive thematic analysis.^30,31^ Transcripts were line-by-line coded by two authors, both female and White British: a senior qualitative researcher (NE); and a doctoral researcher with relevant interview experience (SC). They independently read and coded two transcripts (13%), with discussion to agree the approach. The remaining transcripts were coded and preliminary themes developed by SC, which were discussed frequently with CG, VR, and NE. The resulting themes and subthemes were reviewed and agreed by other authors. NVivo (version 12)^32^ was used for data management and analysis.

## Results

### Sample characteristics

All 15 practitioners were female, which is typical for a female-dominated workforce.^33^ Thirteen were classified as White British and two as Asian British. The mean time for which practitioners had been delivering NHSHCs was 4.7 (±2.4) years (range 9.0 months–9.0 years). Six practitioners had received no formal NHSHC training. Where training was reported (*n* = 9), it was in general delivery and processes.

Mean interview duration was 52.6 (±14.4) min (range 28.8–84.0 min). Analysis produced two main themes relevant to the aim of this article: ‘communicating CVD risk’ (with four subthemes); and ‘understanding CVD risk’ (with two subthemes). However, to make clear the relevance of findings for practice as well as research they are presented by risk calculator rather than by theme. Illustrative quotations are labelled to show the practitioner identifier, risk calculator group, and their role (HCA or GPN). A full report of the results will be available elsewhere.^34^

### Qualitative findings

#### 10-year percentage risk calculator (QRISK2)

Practitioners expressed a degree of confidence in communicating to patients their estimated 10-year risk (QRISK2):

*‘I think I’m confident … I think I deliver it well.’* (9.1, QRISK2, HCA)

However, there was reported variation in its application. Some practitioners said that they delivered CVD risk information to all patients in the same way, ‘*like a robot. I think I say the same thing to every patient’* (6.1, QRISK2, GPN), removing the opportunity to tailor risk communication to individual patient needs and understanding.^35^ Other practitioners said that they adapted delivery:

‘*I try and explain* [10-year risk] *it for the level of the person that is sitting there and adapt it.* *’* (6.2, QRISK2, GPN)

Despite the communication of QRISK2 being mandated in NHSHCs,^36^ there was variation in whether or not practitioners chose to do so:

*‘*[If] *you think the patient perhaps is not going to pay any attention to you, they are not going to take it in, then no* *.* *’* (6.2, QRISK2, GPN)

*‘I personally always do it* *… because the whole point of the health check is that you reach that number* *… ’* (1.1, JBS3, GPN)

Factors influencing whether or not to tell patients their 10-year CVD risk *‘very much depends on the patient’* (6.1, QRISK2, GPN); specifically, their age, perceived ability to understand (owing to *‘* *education* *’* or *‘* *language barrier*[s]*’* [11.1, JBS3, HCA]), or the perceived likelihood of engagement:

*‘... maybe they do understand, but they don’t care* *… so they don’t want to know, they don’t want to discuss it* *… ’* (3.1, QRISK2, HCA)

There was also a suggestion that NHSHCs provided a lot of information for patients to process, which could limit *‘* *whether they’ve taken any of it in* *’* (8.2, JBS3, GPN).

Practitioner perceptions of the usefulness of the 10-year risk score was also important. Several saw the value of 10-year risk in guiding *‘the diagnosis and the referrals’* (2.1, QRISK2+, HCA), but thought that *‘giving them a percentage, doesn’t inspire them, doesn’t motivate them’* (1.2, JBS3, HCA). One practitioner said that they *‘* *don’t always know how helpful it is to patients* *’* (6.2, QRISK2, GPN).

The ways in which practitioners described 10-year risk suggested that it was not used to facilitate discussion of CVD risk with patients. Rather, it was described as *‘a quick go-to tool … it’s OK, it’s all that we have’* (10.1, QRISK2, GPN), which was used to guide clinical decisions by identifying those with elevated risk using thresholds:

*‘... you have got that 10% … you see it and you think about it, “Well they are going to need a statin* *“*.*‘* (6.2*,* QRISK*2,* GPN)

Despite expressed confidence around communicating 10-year risk, practitioners often demonstrated limited understanding and confidence in explaining the score (beyond determining low-, medium-, or high-risk thresholds). This could limit how much practitioners engage patients in further discussion of risk:

*‘I feel confident in the way that I give it … but then you are only reading off a piece of paper … I don’t feel that I understand … what the percentage is really.*’ (1.2, JBS3, HCA)

Others felt that they understood the risk score, but questioned their ability to communicate it:

*‘* *I can probably babble sometimes and think, “Even I didn’t understand that*”.*’* (12.1, QRISK2+, HCA)

Critically, practitioners used patients' verbal and non-verbal reactions to their 10-year risk score to gauge understanding, *‘nodding their head, so I think they all understood what I was trying to say’* (11.1, JBS3, HCA). However, they also recognised the limitation:

*‘It was based on their reactions, but you did wonder sometimes whether they actually fully understood.’* (5.1, JBS3, GPN)

Practitioners expressed a common feeling that even if patients *‘say they understand the percentage, some will, and some won’t’* (12.1, QRISK2+, HCA):

*‘*... *they seemed to* [understand 10-year risk] *… they didn’t say otherwise, but maybe they wouldn’t, I don’t know*.*’* (6.2, QRISK2, GPN)

The lack of confidence in their understanding and that of their patients highlighted a training need, which practitioners recognised:

*‘* *There is definitely room for improvement, I can see myself there.* *’* (6.1, QRISK2, GPN)

Several acknowledged *‘* *we do need more training in* [CVD risk communication]*’* (1.1, JBS3, GPN). For those who had received some training, it was limited, *‘* *can’t even really call it on the job training* *’* (1.1, JBS3, GPN), and *‘* *on how to use the* [point-of-care testing] *machine ... rather than how to talk ... and understand the risks* *’* (6.2, QRISK2, GPN). None reported training in CVD risk understanding or communication.

#### JBS3 risk calculator

##### Event-free survival age

Event-free survival age appeared to be *‘* *the hardest one to try and communicate’* (7.1, JBS3, HCA). Unlike 10-year risk, event-free survival age does not have thresholds that identify patients as low-, medium-, or high-risk, which were relied on as cues for discussion or action:

*‘... there’s no benchmark to give that comparison to be like “right you can live ‘til 84 and so-and-so live till 82”, so that one was harder.’* (7.1, JBS3, HCA)

As illustrated above, some practitioners misinterpreted this lifetime risk metric as expected age of survival (rather than CVD event-free survival), which could result in patients being given incorrect or misleading information. One practitioner also questioned whether event-free survival age would motivate a patient to make a change if the predicted age the patient is expected to live without a CVD event was high:

*‘* *...* *you have told them that they are going* [to] *live until they’re 82 without any heart attacks or strokes* *… Is that motivating them* *… I would say not* *… Because …* *they are going get to 82 without anything happening to them.’* (1.2, JBS3, HCA)

##### Heart age

Heart age was perceived positively. Practitioners were confident in communicating heart age, *‘cos it’s just the easier one to … explain’* (8.2, JBS3, GPN). It has inherent benchmarking through comparison with chronological age, thought to make it easier than 10-year risk for patients to understand, *‘* *they can get their head around that concept of their* [heart] *age a lot better than* [10-year] *risk score’* (7.1, JBS3, HCA):

*‘*... *they know how old they are and then they are exactly …* *“* *oh it’s the same age as me”, so I think they understood that more*.*’* (11.1, JBS3, HCA)

In turn, practitioners perceived that heart age could motivate patients by highlighting the need to make lifestyle changes, and, in particular, they believed that patients liked and responded strongly to the *‘* *visual of heart age* *’* (8.1, JBS3, HCA):

*‘*... *because it is an actual* [heart] *when you go onto that screen of heart age. The heart is there, you can’t escape that and then you have got your age right by it*.’ (8.1, JBS3, HCA)

When discussing their limited understanding of 10-year risk, one practitioner stated *‘* *I prefer the heart age’* (1.2, JBS3, HCA).

##### Risk score manipulation and visual displays

Practitioners were positive about risk factor manipulation and visual displays in JBS3. Those using JBS3 reported that visual features provided an alternative method to communicate risk to patients, who *‘* *said it is quite nice to see visually,* [as] *opposed to me talking’* (8.1, JBS3, GPN). This was thought to improve patient engagement:

*‘* *They actually are interested. They have come closer to me, their body language was good, they were looking at that, they did ask questions and they were happy, because I think it was visualised*.*’* (11.1, JBS3, HCA)

Practitioners perceived that this benefit for patient engagement led to better patient understanding by showing the benefits of reducing risk (gain framing), which might be more appropriate when discussing prevention:

*‘* *I think they understand more … When you show them that if you bring your blood pressure down, your cholesterol down, your weight down … how that can affect the results … they start to think about exercise and lifestyle … it’s like an eye-opener to them*.*’* (4.1, JBS3, HCA)

It was also used to show the consequences of high CVD risk (loss framing), which might be more appropriate for those with a family history of CVD:

*‘*... *when I was increasing the blood pressure, or the cholesterol, or if they were a smoker, they actually saw the difference and then they were like, “no I won't, and thank God I am not* [a smoker]*”, you know so it was a difference on them as well*.*’* (11.1, JBS3, HCA)

Again, the VSR prompted some practitioners to appraise their risk communication; for example, one practitioner described their communication of heart age as *‘* *just sort of abrupt wasn’t it? … There didn’t seem to be much of a consultation around it* *’* (1.2, JBS3, HCA); further evidence of the recognised training need.

## Discussion

### Summary

Data were reported from the first VSR interviews with NHSHC practitioners. The focus was on their understanding, perceptions, and experiences of CVD risk communication in NHSHCs using QRISK2 or JBS3. Despite apparent confidence in delivering the QRISK2 10-year risk scores, they were not well understood by practitioners and were regarded primarily as a means of identifying patients as low-, medium-, or high-risk to guide decisions around routine medical follow-up, rather than as a tool to facilitate a discussion of CVD risk with patients. Ultimately, a lack of understanding and confidence in explaining 10-year risk was observed among NHSHC practitioners.

There was a perception that patients were more responsive to, and, therefore, more likely to display intentions towards risk-reducing behaviours in response to heart age and risk score manipulation in JBS3, and that patients liked the visual displays (including the heart-age image). Some practitioners, however, misunderstood event-free survival age. The lack of thresholds to indicate when risk was ‘high’ (as with 10-year risk) and lack of inherent comparison (as with heart age versus chronological age), limited practitioners’ confidence with event-free survival age.

None of the practitioners had received specific training in risk communication, six had no training at all, and they all recognised this training need.

### Strengths and limitations

The strengths of this study included use of VSR to allow practitioners to reflect on actual events rather than memories (which are subject to recall bias). They were able to reflect on how they felt at that time, and it allowed for specific reflection on language used and nuances (for example, body language and real-time reactions).^27,28^ In addition, the sample included practitioners from general practices in areas that varied in deprivation, and who varied in their role (HCA and GPN) and experience.

Limitations are recognised. First, all practices were based in the West Midlands. It cannot be assumed that practitioners were representative of the wider population who deliver NHSHCs. Second, potential benefits of JBS3 could have been undermined by practitioners’ lack of familiarity and practice using it. However, those who used JBS3 in the RICO study were given a verbal explanation during practice initiation visits, written materials, and a short-video tutorial on how to use JBS3, and were asked to practise using JBS3 in the NHSHC in advance of data collection. Further training was not provided in order to preserve the ecological validity of studying how the tool might be used if made available. Third, staffing changes meant that the two researchers who completed the VSR interviews (VR and LC) were not able to lead the coding and preliminary theme development, but were involved in all subsequent stages.

### Comparison with existing literature

The findings confirm health professionals’ difficulties in explaining percentage CVD risk^6–9^ and suggest reasons for the brevity of CVD risk discussion observed in NHSHCs.^24,25^ In particular, the data support the existing evidence that, often, practitioners do not understand percentage 10-year risk sufficiently well for effective risk communication. This is perhaps not surprising. Such short-term absolute CVD risk metrics were developed to guide clinical decisions and QRISK2 was the standard NICE-recommended tool at the time of this study.^4^ Practitioners demonstrated a concomitant level of understanding; primarily, use of the 10% threshold as a trigger to discuss statins. However, this falls short of the expected level of practitioner understanding for CVD risk communication that can engender patient understanding and inform person-centred risk management discussions.^2,3,36^ This might relate to the lack of specific training in risk communication, which accords with a general training need reported elsewhere.^37,38^ The apparent benefits of some JBS3 functionalities support the suggestion that, while absolute risk should guide clinical decisions,^5^ alternative CVD risk metrics and tools might aid understanding. To address this gap, some general practices (four in RICO) use additional software with further functions, risk scores, and presentations, such as those in JBS3.

Practitioners’ positive perceptions of heart age supports existing literature that suggests it improves comprehension and potential impact. Compared with usual care or alternative risk scores, heart age has been identified as easier to communicate (by practitioners) and easier to recall (by patients).^5,12^ However, there remain questions regarding its ability to improve CVD risk factors and lifestyle change intentions,^13^ and some patients question the credibility of heart age.^17,39,40^ Similar to QRISK2, it is possible that practitioners do not fully understand what heart age means (that is, the estimated age of someone of the same sex and ethnic group, and annual risk of a CVD event, but with optimal risk factors), but it is intuitive that an ‘old heart age’ is an undesirable outcome.^41^ Therefore, unlike QRISK2, a superficial understanding of heart age might be adequate for an informed discussion of CVD risk.

Conversely, practitioners often misinterpreted event-free survival age as predicted age of death. Consequently, event-free survival age was not well-explained to patients during NHSHCs.^25^ A paucity of literature examines this metric, and none that is specific to NHSHCs. A 2011 review of quantitative studies of CVD risk communication strategies concluded that, compared with time frames of >10 years, shorter time frames improved accuracy of perceptions of CVD risk and risk-reducing behavioural intentions.^20^ The data suggest that the lack of intrinsic comparison (as with heart age) or benchmarking as low-, medium-, or high-risk (as with QRISK2), is part of the problem. It prevented a basic interpretation of a risk score as ‘good’ or ‘bad’, ‘low’ or ‘high’, which is possible with heart age and 10-year risk, even without fully understanding the score. Despite the theoretical benefits of lifetime risk over short-term CVD risk estimates, the utility of event-free survival age was undermined through poor understanding.

Risk score manipulation has potential for application in NHSHCs. Videorecorded NHSHCs suggested that it might improve patients’ understanding and appraisal of CVD risk, and improve engagement.^25^ Similarly, interviewed practitioners believed that risk manipulation helped to engage patients in risk discussion and that they understood. However, this was based on their reading of how patients reacted to information. As the authors reported elsewhere,^25^ patient responses to risk information were minimal and participants acknowledged that they did not really know if patients actually understood their risk scores. Literature on interactive graphical risk representations indicates potential benefits for provoking a more emotional response,^42^ but highlights the importance of user competence.^18^ In RICO, there were examples of practitioners manipulating inappropriate risk factors, such as showing a non-smoker with raised cholesterol how their risk would increase if they started smoking, rather than showing the benefit of reducing cholesterol.^25^ Therefore, despite the positive perceptions among practitioners, user competence might have limited the impact.

### Implications for research and practice

Implications for commissioners and deliverers of NHSHCs include the need to realign expectations of CVD risk communication, and the tools and training to support practitioners. Use of QRISK2 10-year CVD risk to guide clinical decisions, rather than for CVD risk communication, is consistent with its original purpose, but not with the expectations of NHSHCs. Heart age and risk manipulation functions of JBS3 should help practitioners to promote patient understanding of CVD risk. However, there is an urgent training need to improve practitioners’ understanding and confidence in communicating CVD risk.

Researchers should explore practitioner competencies and patient needs to inform training. This could include mapping practitioner competencies to NHSHC requirements, and designing and testing training to address the gaps.

## Trial registration number

ISRCTN10443908.

## Data

The data generated by this qualitative study are not suitable for sharing beyond that presented in this report. Most data are in the form of interview transcripts, which present ethical challenges regarding data sharing for secondary analysis; for example, threats to participant anonymity, and unknown future use of data in relation to original participant consent and ethical approvals. For more information, please contact the corresponding author.
